# Significant insights from a National survey in China: PrEP awareness, willingness, uptake, and adherence among YMSM students

**DOI:** 10.1186/s12889-024-18512-y

**Published:** 2024-04-11

**Authors:** Yuanyuan Liu, Yidan Xian, Xuan Liu, Zhaoyu Cheng, Siyue Wei, Jianping Wang, Jiageng Chen, Changping Li, Jun Ma, Jie Yang, Fengli Liu, Maohe Yu, Zhongdan Chen, Zhuang Cui

**Affiliations:** 1 Department of Epidemiology and Biostatistics, School of Public Health, Tianjin Medical University, No. 22 Qixiangtai Road, Heping District, Tianjin, 300070 China; 2 Tianjin Key Laboratory of Environment, Nutrition and Public Health, Tianjin, China; 3 “Shenlan” Public Health Counseling Service Center, Tianjin, China; 4 STD & AIDS Control and Prevention Section, Tianjin Center for Disease Control and Prevention, Tianjin, China; 5 Tianjin Key Laboratory of Pathogenic Microbiology of Infectious Disease, Tianjin Centers for Disease Control and Prevention, Tianjin, 300011 China; 6 HIV/Hepatitis/STI/TB, World Health Organization Representative Office in China, 401 Dongwai Diplomatic Building 23, DongzhimenwaiDajie, Chaoyang District, Beijing, 100600 China

**Keywords:** YMSM, Student, PrEP, Willingness, Uptake, Adherence

## Abstract

**Introduction:**

Few studies focused on the Pre-Exposure Prophylaxis (PrEP) -related aspects, and the applicability of prior evidence to young men who have sex with men (YMSM) students was unknown. This study aimed to assess the awareness, willingness, uptake, and adherence (AWUA) to PrEP among YMSM students in China and to explore the associated factors with these stages.

**Methods:**

A cross-sectional survey with a sizable sample of 1151 was conducted among YMSM students aged 16 and above, who self-identified as men who have sex with men(MSM) and resided in mainland China between October 20 and December 20, 2021. The chi-square test and Fisher’s exact test were used for univariate analysis, followed by multivariable logistic regression analysis of influencing factors at all levels.

**Results:**

According to the cascade analysis approach, 88.71% of the participants were aware of PrEP, among which 66.7% expressed willingness to use it. Among those who were willing to use PrEP, only 13.80% took it, and of those who took it, 44.68% adhered to it. The students taking PrEP were those with higher education (OR = 4.239, 95% CI: 1.334–13.467), residence in pilot cities (OR = 2.791, 95% CI: 1.498–5.198), residence in high-risk areas (OR = 5.082, 95% CI: 2.224–11.612), engagement in multi-person sexual behavior (OR = 2.186, 95% CI: 1.236–3.867), and substance use (OR = 1.908, 95% CI: 1.167–3.118). Furtherly, students with higher adherence to PrEP were likely to have receptive sexual behaviors (OR = 8.702, 95% CI: 2.070-36.592), absence of substance use (OR = 4.468, 95% CI: 1.371–14.561), and uptake of PrEP through daily oral route. (OR = 7.065, 95% CI: 1.699–29.371).

**Conclusion:**

YMSM students exhibit distinct patterns of “high awareness, low willingness, low uptake, and low adherence” to PrEP. Strategies for reduction in the acquisition of HIV prioritizing the current features of utilizing PrEP were urgently warranted.

**Supplementary Information:**

The online version contains supplementary material available at 10.1186/s12889-024-18512-y.

## Introduction

Globally, approximately 4,000 individuals acquire HIV daily, of which over a quarter fall within the young aged 15–24 years old [1]. In the Asia-Pacific region, more than half (52%) of newly transmitted young individuals with HIV are identified as gay men and other men who engage in sexual activities with men [2]. In China, the prevalence of acquiring HIV among men who have sex with men (MSM) stands at 5.4% [3], with approximately 236.5 thousand MSM aged 15–19 and 1.1 million aged 20–24 having acquired HIV [4]. The incidence of newly acquiring HIV among college students has sharply increased from 30 to 50% [5] over the past few years. Yet, research on HIV prevention targeting this particular group remains inadequate.

Pre-exposure Prophylaxis (PrEP) has demonstrated a remarkable efficacy of 99% in reducing the risk of acquiring HIV from sexual intercourse [6]. In 2019, the World Health Organization revised its guidelines to include an event-driven approach as an alternative to the daily oral PrEP regimen for MSM. This approach involves the intake of two tablets orally 2 to 24 h before engaging in high-risk sexual behavior, followed by one tablet every 24 h and 48 h after the initial dose [7]. However, a study conducted in China indicates that a mere 20% of MSM are aware of PrEP, and it is estimated that less than 1% of the MSM population, which amounts to 8.226 million individuals, actually initiate PrEP [8, 9]. A study conducted in Chengdu in 2019 found that awareness of daily oral PrEP and event-driven PrEP was 33.8% and 30.7%, respectively, and the willingness to take daily oral and event-driven PrEP in the next six months was 60.1% and 79.2%, respectively [10].

Given that students are actively engaging in sexual activities, displaying a natural curiosity to explore sexual experiences, and finding themselves at a critical phase of physical and psychological development, it is imperative to develop comprehensive prevention and control strategies that encompass the use of PrEP for young men who have sex with men(YMSM). To make it an effective approach, it is crucial to understand the profile of PrEP utilization and explore the various factors associated with awareness, willingness, uptake, and adherence (AWUA). Previous studies have indicated that factors such as literacy, cost, sexual role, and sexual behavior are interconnected with PrEP. For instance, a study conducted in 2013 by Jose et al. in the United States [11] demonstrated that literacy, residency, and insurance coverage were linked to perceptions surrounding PrEP. Furthermore, a study conducted in California in 2017 [12] revealed that PrEP usage was associated with factors such as income, engaging in receptive condomless anal sex, having HIV-positive sexual partners, substance use, and recent sexually transmitted diseases (STDs). Hence, there is an urgent necessity to comprehend the current state of awareness, attitudes, and practices of YMSM students in China regarding PrEP. The acquirement of such insights will contribute to the provision of evidence for future research endeavors in this domain.

The purpose of this study was to gain a comprehensive understanding of AWUA among YMSM students in China and to explore the factors associated with these aspects to offer insights and recommendations for future interventions and strategies in China. The ultimate goal is to make a significant contribution towards the global elimination of AIDS by the year 2030.

## Methods

### Participants

**Fig. 1:**
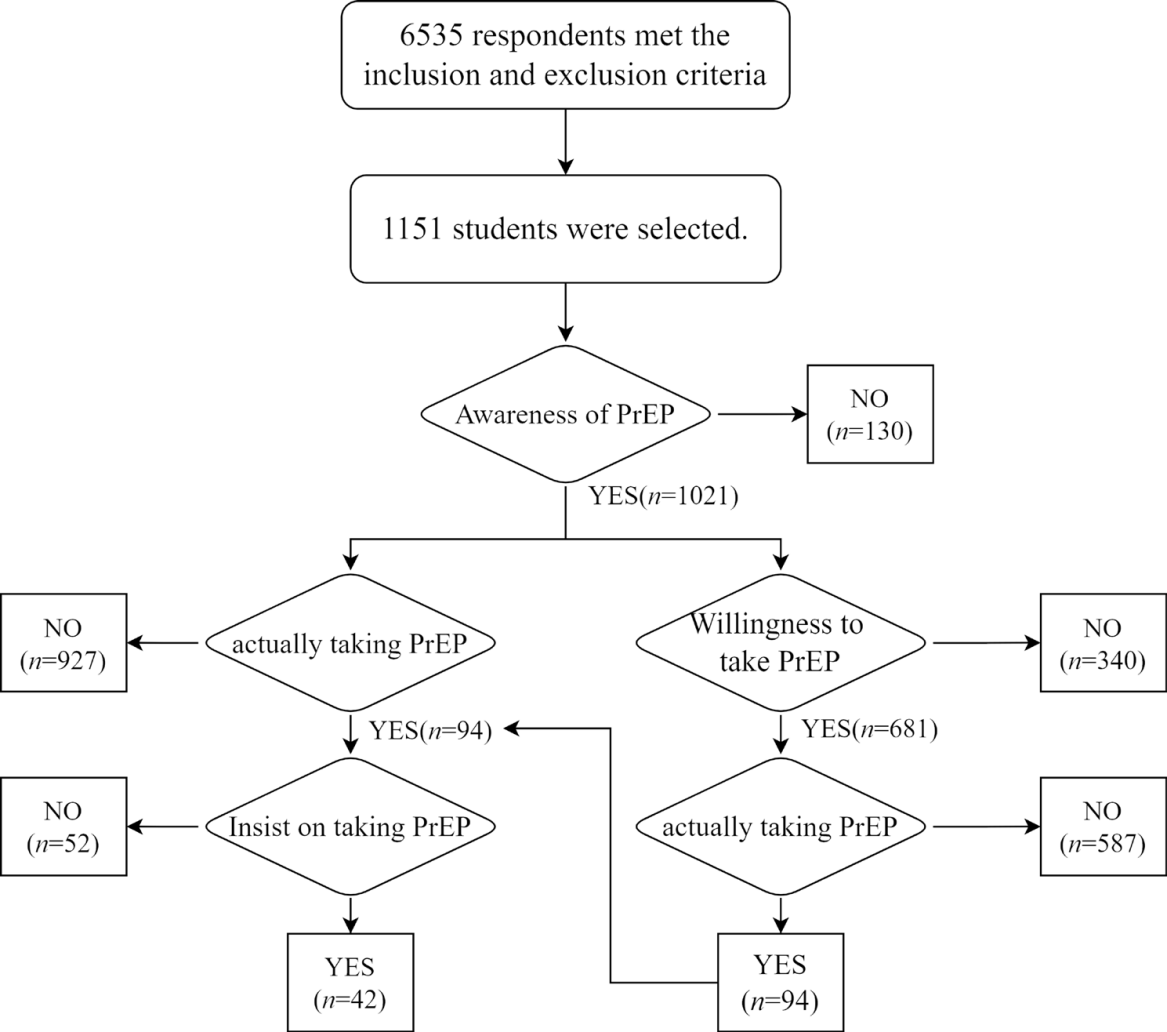
Flowchart illustrating the cascade analysis process from PrEP awareness to adherence

This cross-sectional study, conducted between October 20 and December 20, 2021, examined oral PrEP awareness, willingness, and use among MSM in 31 Chinese regions, comprising 22 provinces, four municipalities, and five autonomous regions. Sponsored by the WHO China Office, it was executed by the Tianjin “Shenlan” Public Health Counselling Service Center, with the assistance of MSM community organizations in distributing electronic questionnaires across geographical areas. For participant recruitment, a combination of online and offline methods was employed using convenience and snowball sampling. The online approach involved community workers publicizing the study and recruiting participants in WeChat Moments and Groups. The offline approach consisted of staff members visiting bars and bathhouses frequented by the gay community to recruit participants. A stratified proportional sampling method was employed to recruit a total of 6535 MSM aged 16 and above, who were confirmed to be HIV negative and self-reported engaging in sexual intercourse with at least one man within the preceding six months. From the initially enrolled participants, a subgroup consisting of 1151 individuals was identified as students. The following flowchart illustrates the cascade analysis process from PrEP awareness to adherence (Fig. 1).

### Measures

The sociodemographic variables captured in this study included age, educational attainment, monthly income, current place of residence, and inquiries concerning sexual conduct within the preceding six months. In accordance with the PrEP cascade framework, the investigation examined PrEP awareness, PrEP willingness, PrEP uptake, and adherence to PrEP protocols. The study examined the rationale behind the decisions of participants who were aware of PrEP yet exhibited reluctance to utilize it, expressed willingness to initiate but had not commenced its use, and those who adhered to or discontinued its usage.

#### PrEP awareness

PrEP awareness was evaluated using “Have you heard of the term ‘PrEP’?” Participants responding affirmatively were classified as aware of PrEP, while those responding negatively were deemed unaware.

#### PrEP willingness

PrEP willingness employed a single-item measure which posed the question, “Would you be willing to utilize ‘PrEP’?” Participants responding positively were categorized as having willingness, whereas those responding negatively were considered lacking willingness.

#### PrEP uptake

The uptake of PrEP was evaluated through a single-item measure: “Have you ever used ‘PrEP’?” Participants responding positively were recognized as having ever utilized it, whereas those responding negatively were regarded as non-users.

#### Adherence to PrEP

For daily users, adherent individuals were defined as those who remained consistent in taking PrEP daily or more than four capsules per week. For event-driven users, adherence was determined by their insistence on completing the 2-1-1 regimen on time.

#### Definition of relevant variables

**Educational attainment** The number of years studied in each category of “educational attainment”: Senior high or secondary school: 9–12 years; Junior college or College graduate: 13-16years; Graduate degree or higher: 17 years or above.

**STD** Defined as responding in the questionnaire: “In the past year, have you ever been diagnosed with any of the following sexually transmitted diseases (STDs)? These STDs include: syphilis, gonorrhea, condyloma acuminata, genital herpes, genital tract Chlamydia trachomatis acquisition, and others.”

**Substance use in the preceding 6 months** Defined as responding in the questionnaire: “In the past six months, have you used any of the following substances during same-sex sexual activity? These substances include Viagra, rush, 0 capsules, G-spot liquid, and others.”

**Frequency of condom use in anal sex during the preceding 6 months** In the questionnaire, individuals’ condom use was categorized based on their frequency of use during their most recent anal intercourse experience. The original categories include “Never” “Occasionally,” “Frequently,” and “Always.” For analysis, this variable was reclassified as follows: Low frequency that included those who “Never” and “Occasionally,” while high frequency encompasses “Frequently” and “Always.”

**Regional Division** Gross Domestic Product (GDP) is an indicator of a region’s economic condition and level of development. According to the 2021 annual report of the National Bureau of Statistics, the top 10 provinces and cities with the highest GDP per capita are classified as regions with high GDP, the bottom 10 provinces are classified as regions with low GDP, and the rest are classified as regions with medium GDP [13]. Yunnan and Sichuan were identified as high-risk regions based on existing research [14], while Shenyang, Beijing, Shenzhen, and Chongqing were selected as pilot cities [15].

**Ways of oral PrEP** Based on the answers in the questionnaire, the dosing patterns were categorized as “daily oral PrEP”, “ED-PrEP”, and“both”. Among them, “both” means that both ways of taking have been used, including the situation of “daily oral PrEP” to “ED-PrEP” or “ED-PrEP” to “daily oral PrEP”.

### Data analysis

In this research, a cascade approach was employed to systematically analyze the PrEP awareness, willingness, uptake and adherence. Quantitative data were presented using frequency and percentage values (n (%)). At the same time, the Chi-square test and Fisher’s exact test were utilized to assess differences in demographic characteristics and sexual behavior patterns between different groups. Variables with a significance level of *P* < 0.05 in the univariate analysis were subsequently included in a multivariable logistic regression model, and the final correlates were determined using a stepwise regression process. All statistical analyses were performed using SAS software, version 9.4 (SAS Institute, Cary, NC, USA), with a significance threshold set at *P* < 0.05. Forest plots were generated using the package “forestploter” in R software (4.1.3). In all analyses, two-sided *P* < 0.05 was defined as significantly different.

## Result

### Participants’ characteristics and PrEP current status

The study sample consisted a total of 1151 YMSM students, and a comprehensive overview of their socio-demographic and behavioral characteristics is presented in Table 1. Most of the participants were aged 16–24 (89.8%), with 75.6% having obtained a university-level education or higher. Furthermore, 71.2% of participants reported having no fixed income. Among the individuals who utilized PrEP, 20.2% followed the “daily oral PrEP” regimen, while 51.1% employed an “event-driven PrEP” approach.

**Table 1:** Demographic characteristics and sexual behavior patterns of MSM students who have knowledge about PrEP, n (%)

Features	Total population (n = 1151)	Unawareness of PrEP (n = 130)	Awareness of PrEP (n = 1021)	Wald χ^2^	*P*-value
**Age**				3.668	0.0555^*^
16∼	1034 (89.8)	123 (94.6)	911 (89.2)		
25∼	117 (10.2)	7 (5.4)	110 (10.8)		
**Educational attainment**				2.117	0.3470^*^
Senior high or secondary school	88 (7.6)	10 (7.7)	78 (7.6)		
Junior college or College graduate	870 (75.6)	104 (80.0)	766 (75.0)		
Graduate degree or higher	193 (16.8)	16 (12.3)	177 (17.3)		
**High-risk areas**				1.265	0.2607^*^
No	1092 (94.9)	126 (96.9)	966 (94.6)		
Yes	59 (5.1)	4 (3.1)	55 (5.4)		
**Pilot cities**				0.108	0.7421^*^
No	1063 (92.4)	121 (93.1)	942 (92.3)		
Yes	88 (7.6)	9 (6.9)	79 (7.7)		
**Economic level division**				0.975	0.6142^*^
High GDP	513 (44.6)	53 (40.8)	460 (45.1)		
Medium GDP	384 (33.4)	45 (34.6)	339 (33.2)		
Low GDP	254 (22.1)	32 (24.6)	222 (21.7)		
**Commercial sexual behavior**				1.721	0.1896^*^
No	1053 (91.5)	115 (88.5)	938 (91.9)		
Yes	98 (8.5)	15 (11.5)	83 (8.1)		
**Monthly income (CNY)**				5.022	0.2851^*^
No regular source of income	819 (71.2)	100 (76.9)	719 (70.4)		
<3000 RMB	222 (19.3)	16 (12.3)	206 (20.2)		
3000–5000	75 (6.5)	10 (7.7)	65 (6.4)		
5000–8000	22 (1.9)	2 (1.5)	20 (2.0)		
8000∼	13 (1.1)	2 (1.5)	11 (1.1)		
**Results of the most recent HIV test during the preceding 6 months**				26.055	< 0.0001^**^
Never	124 (10.8)	31 (23.8)	93 (9.1)		
Negative	1027 (89.2)	99 (76.2)	928 (90.9)		
**STD**					
No	1060 (92.1)	115 (88.5)	945 (92.6)	2.656	0.1032^*^
Yes	91(7.9)	15(11.5)	76(7.4)		
**Sexual role with a man during the preceding 6 months**				5.477	0.1400^*^
Top	318 (27.6)	35 (26.9)	283 (27.7)		
Versatile	217 (18.9)	17 (13.1)	200 (19.6)		
Bottom	505 (43.9)	60 (46.2)	445 (43.6)		
Oral	111 (9.6)	18 (13.8)	93 (9.1)		
**Frequency of condom use in anal sex during the preceding 6 months** ^**f**^				9.235	0.0024^*^
Low	314(27.3)	50 (38.5)	264 (25.9)		
High	837(72.7)	80 (61.5)	757 (74.1)		
**Number of sexual partners during the preceding 6 months**				2.899	0.2347^*^
1–5	1023(88.88)	120(92.30)	903(88.44)		
6–10	87(7.56)	5(3.85)	82(8.03)		
11∼	41(3.56)	5(3.85)	36(3.53)		
**Multi-person sexual behavior**				0.464	0.4959^*^
No	1015 (88.2)	117 (90.0)	898 (88.0)		
Yes	136 (11.8)	13 (10.0)	123 (12.0)		
**Knowledge of HIV acquisition status of sexual partners in the last six months**				9.758	0.0076^*^
Full	565 (49.1)	54 (41.5)	511 (50.0)		
Partial	387 (33.6)	41 (31.5)	346 (33.9)		
No	199 (17.3)	35 (26.9)	164 (16.1)		
**Substance use in the preceding 6 months**				0.313	0.5759^*^
No	709 (61.6)	83 (63.8)	626 (61.3)		
Yes	442(38.4)	47(36.2)	395(38.7)		

*Note* *: χ^2^ test

**Fig. 2:**
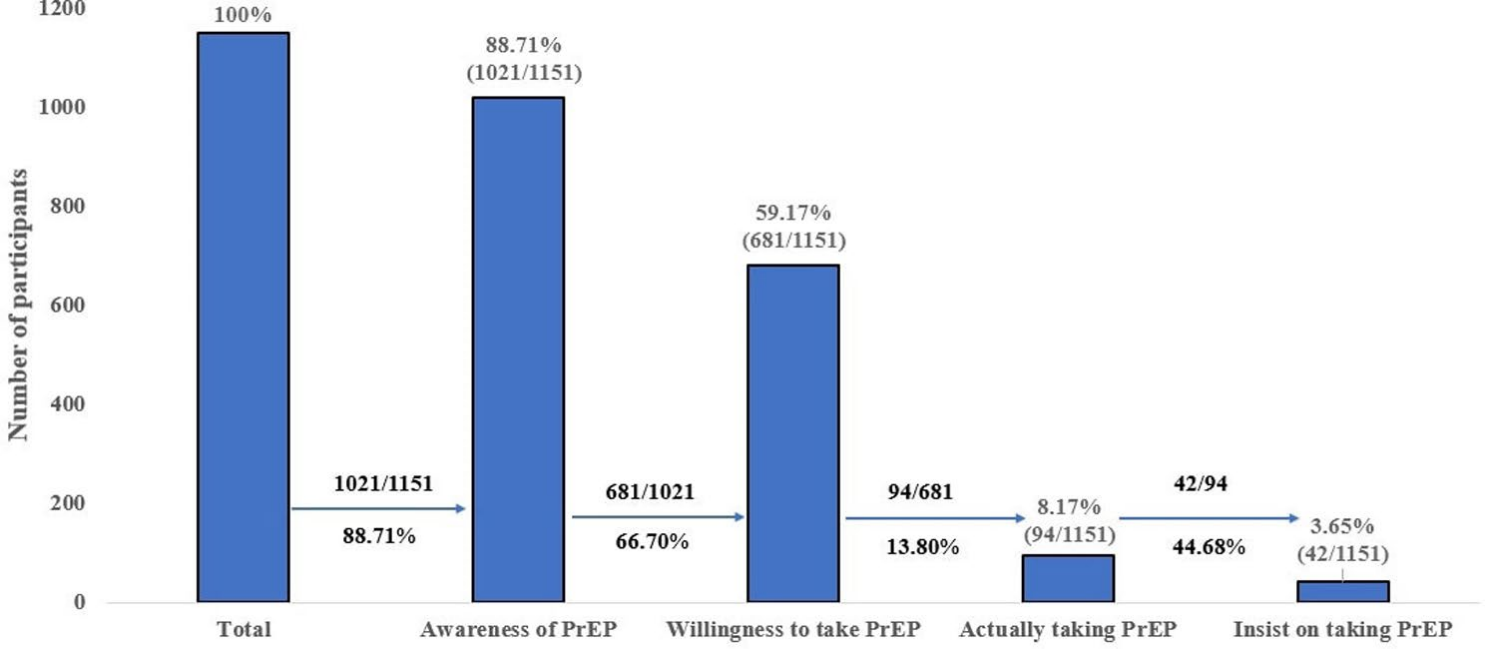
The PrEP cascade

88.71% (1021/1151) were aware of PrEP, 59.17%(681/1151) were willing to take PrEP, while 66.70% (681/1021) of those aware of PrEP were willing to take it; 8.17% (94/1151) actually took PrEP, while 13.80% (94/681) of those willing to take PrEP actually took it; 3.65% (42/1151) adhered to taking PrEP, while 44.68% (42/94) of those who actually took PrEP adhered to it, as shown in Fig. 2.

### Correlations between PrEP-related cognitions and behaviors

**Fig. 3:**
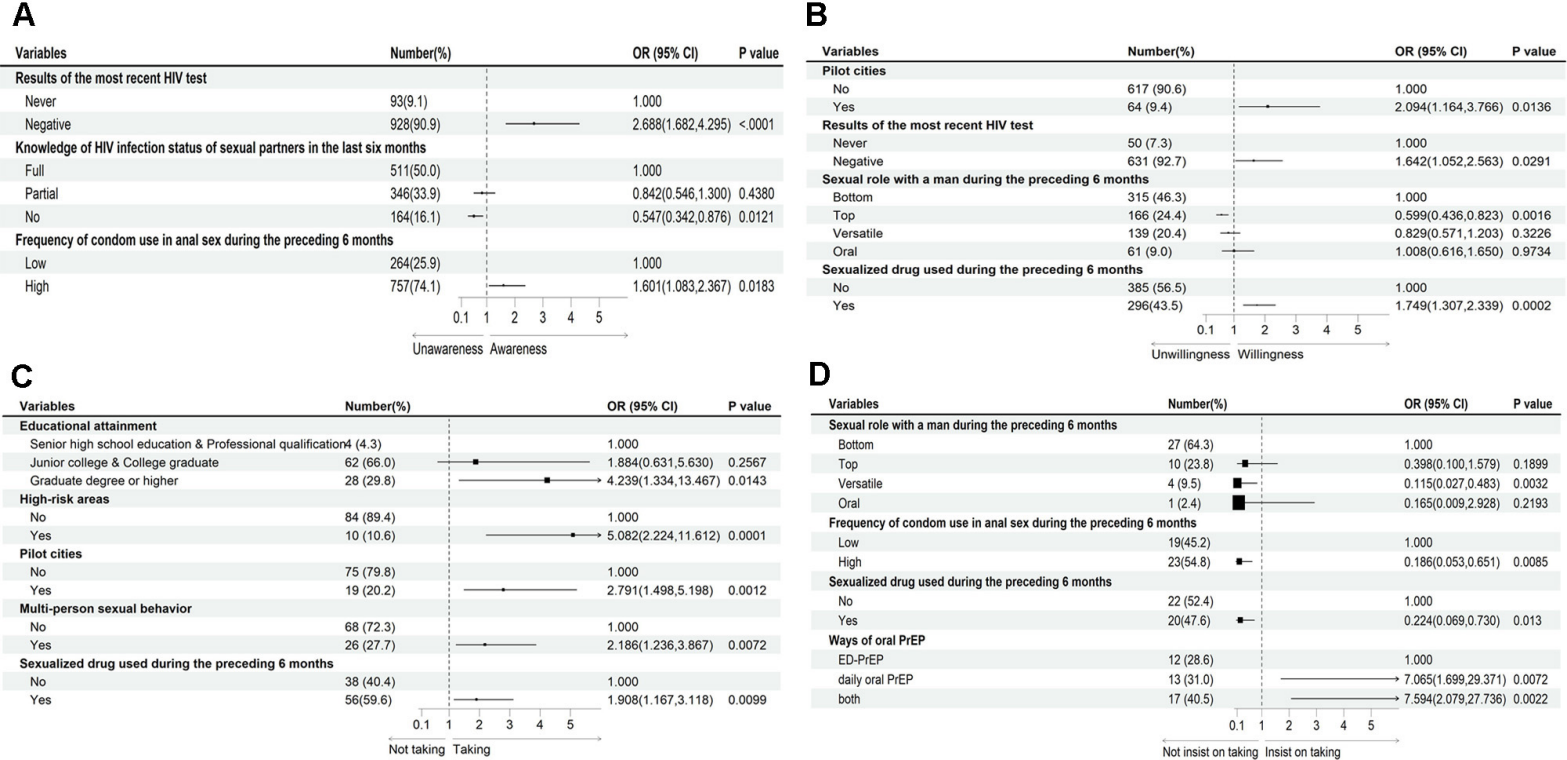
Forest plot (multivariable analysis results). *Note***A**: Those who know about PrEP; **B**: Those who want to take PrEP; **C**: Those who have actually taken PrEP; **D**: Those who insist on taking PrEP

The study findings are presented in Table 1 and Appendices Tables 1, 2 and 3, as well as Fig. 3, which provide comprehensive insights into single and multiple factors. The multivariable analysis revealed that individuals who had recently tested negative for HIV (OR: 2.688, 95% CI: 1.682–4.295) and those who consistently practiced high condom use (OR: 1.601, 95% CI: 1.083–2.367) were more likely to be aware of PrEP. Conversely, participants who reported lacking understanding of their recent partner’s HIV status exhibited lower awareness of PrEP compared to those with complete knowledge (OR: 0.547, 95% CI: 0.342–0.876) (Appendices Table 4).

Furthermore, individuals residing in the designated pilot cities (Beijing, Shenyang, Shenzhen, and Chongqing) (OR: 2.094, 95% CI: 1.164–3.766), those who had received a negative result on their most recent HIV test (OR: 1.642, 95% CI: 1.052–2.563), and those who reported substance use during sexual encounters (OR: 1.749, 95% CI: 1.307–2.339) were more inclined to express willingness to take PrEP. Conversely, individuals who identified themselves as having a “top” sexual role exhibited a decreased likelihood of being willing to take PrEP (OR: 0.599, 95% CI: 0.436–0.823) (Appendices Table 5).

Participants with a “Graduate degree or higher” education (OR: 4.239, 95% CI: 1.334–13.467), those residing in high-risk areas (Yunnan, Sichuan) (OR: 5.082, 95% CI: 2.224–11.612) or the designated pilot cities (OR: 2.791, 95% CI: 1.498–5.198), engaging in multi-person sexual behavior (OR: 2.186, 95% CI: 1.236–3.867), and having experience of substance use within the previous 6 months (OR: 1.908, 95% CI: 1.167–3.118) were more likely to take PrEP (Appendices Table 6).

Regarding adherence to PrEP, individuals who adhered to a “daily oral PrEP” regimen (OR: 7.065, 95% CI: 1.699–29.371) or used “both” (OR: 7.594, 95% CI: 2.079–27.736) were more likely to maintain consistent usage. Conversely, participants who identified themselves as having a “Versatile” sexual role (OR: 0.115, 95% CI: 0.027–0.483), those who reported a high frequency of condom use (OR: 0.186, 95% CI: 0.053–0.651), and those who reported substance use (OR: 0.224, 95% CI: 0.069–0.730) exhibited a lower likelihood of consistent PrEP usage (Appendices Table 7).

### Barriers to PrEP uptake and adherence

**Fig. 4:**
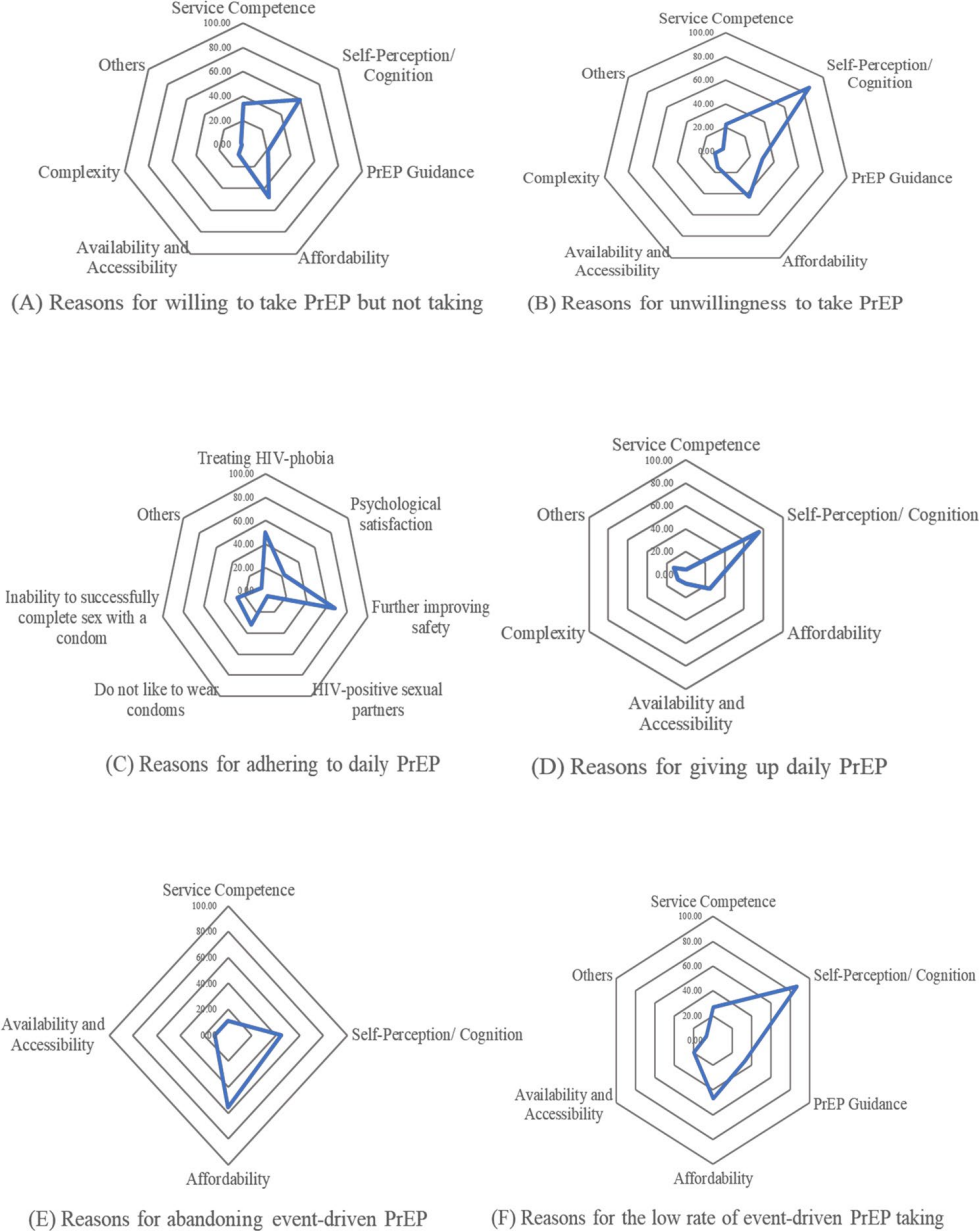
Radar chart (Barriers of PrEP uptake and adherence)

Among individuals who were aware of PrEP (Fig. 4A), the predominant factors contributing to their unwillingness to use it were related to self-perception/cognition (85.88%), affordability (42.94%), and PrEP guidance (30.29%). The predominant barriers hindering PrEP uptake among the YMSM student population (Fig. 4B) were associated with self-perception/cognition (59.80%), affordability (48.04%), and service competence (33.56%). Factors contributing to poor adherence to PrEP (Fig. 4C) were primarily linked to self-perception/cognition (70.45%) and affordability (36.36%).

Given the observation of higher adherence among participants reporting “daily oral PrEP” usage, an examination of their adherence reasons (Fig. 4D) revealed a focus on Further improving safety (68.18%), Treating HIV-phobia (50.00%), and Disinclination to use condoms (31.82%). More comprehensive details can be found in Fig. 4.

## Discussion

PrEP has been shown to be effective in reducing new HIV acquisition in MSM. In the context of the increasing the acquisition of HIV in the YMSM student population, this study investigated PrEP-related profiles and associated factors in YMSM students, with the goal of providing evidence for the next step in proposing prevention and control strategies and measures for this group. To the best of our knowledge, this study represents the first comprehensive cascade analysis of PrEP awareness, willingness, uptake, and adherence, involving a substantial sample size of 1,151 participants from 31 regions across mainland China.

The current YMSM student population exhibits “high awareness, low willingness, low use, and low adherence” regarding PrEP. Specially, the awareness of PrEP among YMSM students was found to be 88.7%, which is higher than that in previous studies [16–18] (ranging from 27.9 to 50%). Additionally, willingness to use PrEP among YMSM students, who were aware of it, was 66.70%, surpassing the willingness reported in previous studies [16–18] (ranging from 57.9 to 65.8%). These results indicated that the awareness and willingness of YMSM students to take PrEP were more prominent compared to the general population. However, it is important to note that the willingness to use PrEP among YMSM students did not correspond proportionally to their awareness. The main obstacle faced by this population appeared to be their low willingness to take PrEP, which was related to factors such as the high cost of the medication. It was evident that intention-behavior gap, namely intentions do not always translate into action, exist for PrEP in YMSM students. A study conducted in Belgium [19] demonstrated that only 30.7% of individuals who expressed willingness to take PrEP actually follow through with it. In contrast, our study found that only 13.80% (94/681) made it. Furthermore, among those who took PrEP, our study revealed that 44.68% (42/94) adhered to the medication, which was lower than adherence rates reported in other studies, especially in the United States [12, 20] where figures were around 70%. Similar findings [21, 22] were observed in previous studies on YMSM groups, which indicated poorer adherence when compared to other MSM populations. Considering that the student group in our study primarily consisted of individuals aged 16–24 years, this study was consistent with previous studies.

The significant intention-behavior gap may have contributed to the escalation rate of acquiring HIV observed among the current population of YMSM students. Consequently, future strategies aimed at preventing and controlling HIV transmission among the YMSM population should prioritize the enhancement of intention-behavior conversion. A major barrier to PrEP utilization among YMSM individuals was the financial burden associated with its use, as indicated by previous studies [23]. Since most students relied on limited financial resources for their living expenses, opting to use PrEP posed a considerable financial challenge for this demographic. Furthermore, our study findings revealed that individuals residing in pilot cities exhibited greater willingness to use PrEP and actually utilized it, as depicted by Fig. 3. This might potentially be attributed to the targeted PrEP promotion efforts in these localities, resulting in heightened awareness and access to PrEP at no cost. To exploit the preventive benefits offered by PrEP in HIV transmission, we strongly advocated for improved accessibility to PrEP among key populations by either reducing the cost of the medication or providing it free of charge.

The multifactorial analysis conducted in this study (Fig. 3) identified several associated factors within the cognitive-belief-behavioral chain [24] including region, HIV testing, sexual behavior characteristics, and the method of PrEP administration. Test takers who underwent HIV testing had access to more accurate information, leading to increased awareness and willingness to use PrEP. These findings aligned with previous studies [25], emphasizing the importance of promoting HIV testing among YMSM students to enhance their knowledge and motivation to take preventive action. Furthermore, individuals who were “fully” aware of their partner’s HIV status exhibited greater concern for personal protection and demonstrated higher health awareness, thereby leading to increased awareness of PrEP compared to those who were “not” aware. Regarding to sexual behavior, the study revealed that individuals engaging in multi-person sexual behavior were more likely to take PrEP, while those assuming a “bottom” sexual role in anal sex exhibit higher adherence to the medication. Additionally, individuals who reported substance use were more likely to use PrEP but displayed lower adherence rates. This may be attributed to a heightened perceived risk of acquiring HIV among this particular population. Notably, a qualitative study [26] uncovered that substance-dependent individuals often engaged in harmful behaviors that hindered their ability to prioritize personal health and maintain proper adherence to PrEP. Furthermore, the study identified lower adherence to PrEP among condom users, indicating a potential relationship with condom self-efficacy. Hence, it was imperative to provide differentiated and targeted services tailored to individuals from diverse regions and at different stages of PrEP use. Taking tailored measures at each stage of PrEP implementation was crucial to ensure not only “high awareness” but also “high uptake and adherence” among the target population.

In order to promote the widespread adoption of PrEP, it was crucial to address the issue of adherence. The “daily oral PrEP” and “both” dosing regimens exhibited higher adherence rates compared to the “event-driven PrEP” dosing method. This finding can be related to the simplicity of the “daily oral PrEP” regimen, which was less prone to forgetfulness and did not require the timing of sexual encounters to be pre-planned, facilitating a more deliberate decision-making process. On the other hand, the “both” approach offered increased flexibility and, consequently, was easier to adhere to. Although prior studies [27, 28] indicated an inclination among MSM populations to prefer an as-needed PrEP regimen rather than a daily one, it was important to note that the efficacy of PrEP was contingent upon consistent adherence. Therefore, it was encouraged for users to prioritize the more adherable “daily oral PrEP” regimen, or consider adjusting their dosage regimen based on their specific circumstances in order to improve adherence.

The concept of the PrEP continuum of care is gaining acceptability within the scientific community in the field of HIV [29]. This study seeks to concretize the implementation of PrEP by exploring the awareness, willingness, uptake, and adherence of PrEP among student YMSM. Specifically, it seeks to enhance the awareness and willingness of MSM to use PrEP, ensure the accessibility of PrEP, and promote adherence to the regimen, thereby refining the concept of “PrEP continuum of care,” which holds significant implications for HIV prevention and control. Based on the above, in conjunction with the concept of tertiary prevention and the study findings, it is essential to not only emphasize increasing awareness and willingness to use PrEP, but also to prioritize its utilization. Furthermore, by addressing the specific barriers identified at each stage of the study, targeted interventions can be developed to enhance PrEP utilization among Chinese MSM, ultimately contributing to the reduction of acquiring HIV rates and the global goal of eradicating HIV by 2030.

This study has some limitations. Firstly, on the one hand, it was a cross-sectional study, and the causal sequence between high-risk behaviors and PrEP use could not be determined. A follow-up study could have been conducted later to address this deficiency. On the other hand, the results of this survey were based on self-reported data and may have been subject to some social desirability bias and recall bias.

## Conclusion

In summary, the YMSM student groups exhibit distinctive attributes, characterized by “heightened awareness, limited willingness, less usage, and low adherence” in relation to PrEP. It is crucial to design interventions tailored specifically to the characteristics of this group. Regarding the implementation of PrEP, targeted approaches can be adopted to enhance its utilization by addressing the factors influencing each stage. These efforts hold significant importance in the exploration of HIV prevention strategies suited for YMSM students.

### Electronic supplementary material

Below is the link to the electronic supplementary material.

Supplementary Material 1

## Data Availability

The datasets used and analyzed during the current study are available from the corresponding author upon reasonable request.
