# Supplementary material for: Significant insights from a National survey in China: PrEP awareness, willingness, uptake, and adherence among YMSM students
Source: BMC Public Health. 2024 Apr 11;24:1009. doi: 10.1186/s12889-024-18512-y (PMC11010336; doi:10.1186/s12889-024-18512-y)
Supplement: Supplementary file 1 — Supplementary Material 1 [file 12889_2024_18512_MOESM1_ESM.docx]

Table S1 Demographic characteristics and sexual behavior patterns of willingness among awareness of PrEP, n (%)

| **Features** | **Awareness of PrEP**  **(n=1021)** | **unwillingness to use PrEP**  **(n=340)** | **Willingness to use PrEP**  **(n=681)** | **Wald *χ^2^*** | ***P*-value** |
| --- | --- | --- | --- | --- | --- |
| **age** | | | | 0.318 | 0.5731^*^ |
| 16~ | 911 (89.2) | 306 (90.0) | 605 (88.8) |  |  |
| 25~ | 110 (10.8) | 34 (10.0) | 76 (11.2) |  |  |
| **Educational attainment** | | | | 0.506 | 0.7765^*^ |
| Senior high or secondary school | 78 (7.6) | 27 (7.9) | 51 (7.5) |  |  |
| Junior college or College graduate | 766 (75.0) | 258 (75.9) | 508 (74.6) |  |  |
| Graduate degree or higher | 177 (17.3) | 55 (16.2) | 122 (17.9) |  |  |
| **High-risk areas** | | | | 1.175 | 0.2785^*^ |
| No | 966 (94.6) | 318 (93.5) | 648 (95.2) |  |  |
| Yes | 55 (5.4) | 22 (6.5) | 33 (4.8) |  |  |
| **Pilot cities** | | | | 7.898 | 0.0049^*^ |
| No | 942 (92.3) | 325 (95.6) | 617 (90.6) |  |  |
| Yes | 79 (7.7) | 15 (4.4) | 64 (9.4) |  |  |
| **Economic level division** | | | | 7.023 | 0.0298^*^ |
| High GDP | 460 (45.1) | 173 (50.9) | 287 (42.1) |  |  |
| Medium GDP | 339 (33.2) | 100 (29.4) | 239 (35.1) |  |  |
| Low GDP | 222 (21.7) | 67 (19.7) | 155 (22.8) |  |  |
| **Commercial sexual behavior** | | | | 6.684 | 0.0097^*^ |
| No | 938 (91.9) | 323 (95.0) | 615 (90.3) |  |  |
| Yes | 83 (8.1) | 17 (5.0) | 66 (9.7) |  |  |
| **Monthly income(CNY)** | | | | 2.653 | 0.6174^*^ |
| No regular source of income | 719 (70.4) | 235 (69.1) | 484 (71.1) |  |  |
| <3000 RMB | 206 (20.2) | 77 (22.6) | 129 (18.9) |  |  |
| 3000-5000 | 65 (6.4) | 20 (5.9) | 45 (6.6) |  |  |
| 5000-8000 | 20 (2.0) | 5 (1.5) | 15 (2.2) |  |  |
| 8000~ | 11 (1.1) | 3 (0.9) | 8 (1.2) |  |  |
| **Results of the most recent HIV test during the preceding 6 months** | | | | 7.709 | 0.0055^*^ |
| Never | 93 (9.1) | 43 (12.6) | 50 (7.3) |  |  |
| Negative | 928 (90.9) | 297 (87.4) | 631 (92.7) |  |  |
| **STD** | | | |  |  |
| No | 945 (92.6) | 323 (95.0) | 622 (91.3) | 4.418 | 0.0356^*^ |
| Yes | 76(7.4) | 17(5.0) | 59(8.7) |  |  |
| **Sexual role with a man during the preceding 6 months** | | | | 12.345 | 0.0063^*^ |
| Top | 283 (27.7) | 117 (34.4) | 166 (24.4) |  |  |
| Versatile | 200 (19.6) | 61 (17.9) | 139 (20.4) |  |  |
| Bottom | 445 (43.6) | 130 (38.2) | 315 (46.3) |  |  |
| Oral | 93 (9.1) | 32 (9.4) | 61 (9.0) |  |  |
| **Frequency of condom use in anal sex during the preceding 6 months** | | | | 0.804 | 0.3698^*^ |
| Low | 264 (25.9) | 82 (24.1) | 182 (26.7) |  |  |
| High | 757 (74.1) | 258 (75.9) | 499 (73.3) |  |  |
| **Number of sexual partners during the preceding 6 months** | | | | 5.556 | 0.0621^*^ |
| 1-5 | 903 (88.4) | 312 (91.8) | 591 (86.8) |  |  |
| 6-10 | 82 (8.0) | 20 (5.9) | 62 (9.1) |  |  |
| 11~ | 36 (3.5) | 8 (2.4) | 28 (4.1) |  |  |
| **Multi-person sexual behavior** | | | | 5.953 | 0.0147^*^ |
| No | 898 (88.0) | 311 (91.5) | 587 (86.2) |  |  |
| Yes | 123 (12.0) | 29 (8.5) | 94 (13.8) |  |  |
| **Knowledge of HIV acquisition status of sexual partners in the last six months** | | | | 9.141 | 0.0104^*^ |
| Full | 511 (50.0) | 189 (55.6) | 322 (47.3) |  |  |
| Partial | 346 (33.9) | 94 (27.6) | 252 (37.0) |  |  |
| No | 164 (16.1) | 57 (16.8) | 107 (15.7) |  |  |
| **Substance use in the preceding 6 months** | | | | 19.681 | <0.0001^*^ |
| No | 626 (61.3) | 241 (70.9) | 385 (56.5) |  |  |
| Yes | 395(38.7) | 99(29.1) | 296(43.5) |  |  |

Note: *: χ^2^ test;

Table S2 Demographic characteristics and sexual behavior patterns of users among willingness of PrEP, n (%)

| **Features** | **Willingness to use PrEP**  **(n=681)** | **No taking PrEP**  **(n=587)** | **Used PrEP**  **(n=94)** | **Wald *χ^2^*** | ***P*-value** |
| --- | --- | --- | --- | --- | --- |
| **age** | | | | 5.275 | 0.0216^*^ |
| 16~ | 605 (88.8) | 528 (89.9) | 77 (81.9) |  |  |
| 25~ | 76 (11.2) | 59 (10.1) | 17 (18.1) |  |  |
| **Educational attainment** | | | | 11.194 | 0.0037^*^ |
| Senior high or secondary school | 51 (7.5) | 47 (8.0) | 4 (4.3) |  |  |
| Junior college or College graduate | 508 (74.6) | 446 (76.0) | 62 (66.0) |  |  |
| Graduate degree or higher | 122 (17.9) | 94 (16.0) | 28 (29.8) |  |  |
| **High-risk areas** | | | |  | 0.0159^**^ |
| No | 648 (95.2) | 564 (96.1) | 84 (89.4) |  |  |
| Yes | 33 (4.8) | 23 (3.9) | 10 (10.6) |  |  |
| **Pilot cities** | | | | 14.980 | 0.0001^*^ |
| No | 617 (90.6) | 542 (92.3) | 75 (79.8) |  |  |
| Yes | 64 (9.4) | 45 (7.7) | 19 (20.2) |  |  |
| **Economic level division** | | | | 1.617 | 0.4455^*^ |
| High GDP | 287 (42.1) | 247 (42.1) | 40 (42.6) |  |  |
| Medium GDP | 239 (35.1) | 202 (34.4) | 37 (39.4) |  |  |
| Low GDP | 155 (22.8) | 138 (23.5) | 17 (18.1) |  |  |
| **Commercial sexual behavior** | | | | 6.694 | 0.0097^*^ |
| No | 615 (90.3) | 537 (91.5) | 78 (83.0) |  |  |
| Yes | 66 (9.7) | 50 (8.5) | 16 (17.0) |  |  |
| **Monthly income(CNY)** | | | | 4.906 | 0.2971^*^ |
| No regular source of income | 484 (71.1) | 424 (72.2) | 60 (63.8) |  |  |
| <3000 RMB | 129 (18.9) | 110 (18.7) | 19 (20.2) |  |  |
| 3000-5000 | 45 (6.6) | 35 (6.0) | 10 (10.6) |  |  |
| 5000-8000 | 15 (2.2) | 12 (2.0) | 3 (3.2) |  |  |
| 8000~ | 8 (1.2) | 6 (1.0) | 2 (2.1) |  |  |
| **Results of the most recent HIV test during the preceding 6 months** | | | | 1.527 | 0.2165^*^ |
| Never | 50 (7.3) | 46 (7.8) | 4 (4.3) |  |  |
| Negative | 631 (92.7) | 541 (92.2) | 90 (95.7) |  |  |
| **STD** | | | | 0.537 | 0.4635^*^ |
| No | 622 (91.3) | 538 (91.7) | 84 (89.4) |  |  |
| Yes | 59(8.7) | 49(8.3) | 10(10.6) |  |  |
| **Sexual role with a man during the preceding 6 months** | | | | 5.443 | 0.1421^*^ |
| Top | 166 (24.4) | 145 (24.7) | 21 (22.3) |  |  |
| Versatile | 139 (20.4) | 116 (19.8) | 23 (24.5) |  |  |
| Bottom | 315 (46.3) | 268 (45.7) | 47 (50.0) |  |  |
| Oral | 61 (9.0) | 58 (9.9) | 3 (3.2) |  |  |
| **Frequency of condom use in anal sex during the preceding 6 months** | | | | 1.500 | 0.2207^*^ |
| Low | 182(26.7) | 152(25.9) | 30(31.9) |  |  |
| High | 499(73.3) | 435(74.1) | 64(68.1) |  |  |
| **Number of sexual partners during the preceding 6 months** | | | | 4.201 | 0.1224^*^ |
| 1-5 | 591 (86.8) | 515 (87.7) | 76 (80.9) |  |  |
| 6-10 | 62 (9.1) | 51 (8.7) | 11 (11.7) |  |  |
| 11~ | 28 (4.1) | 21 (3.6) | 7 (7.4) |  |  |
| **Multi-person sexual behavior** | | | | 17.598 | <.0001^*^ |
| No | 587 (86.2) | 519 (88.4) | 68 (72.3) |  |  |
| Yes | 94 (13.8) | 68 (11.6) | 26 (27.7) |  |  |
| **Knowledge of HIV acquisition status of sexual partners in the last six months** | | | | 0.774 | 0.6790^*^ |
| Full | 322 (47.3) | 277 (47.2) | 45 (47.9) |  |  |
| Partial | 252 (37.0) | 215 (36.6) | 37 (39.4) |  |  |
| No | 107 (15.7) | 95 (16.2) | 12 (12.8) |  |  |
| **Substance use in the preceding 6 months** | | | | 11.516 | 0.0007^*^ |
| No | 385 (56.5) | 347 (59.1) | 38 (40.4) |  |  |
| Yes | 296(43.5) | 240(40.9) | 56(59.6) |  |  |

Note: *:χ^2^ test; **: Fisher's test

Table S3 Demographic characteristics and sexual behavior patterns of adherents among PrEP users, n (%)

| **Features** | **Used PrEP**  **(n=94)** | **Failure to adhere to PrEP**  **(n=52)** | **Stick with PrEP**  **(n=42)** | **Wald *χ^2^*** | ***P*-value** |  |
| --- | --- | --- | --- | --- | --- | --- |
| **age** | | | | 1.679 | 0.1950^*^ |  |
| 16~ | 77 (81.9) | 45 (86.5) | 32 (76.2) |  |  |  |
| 25~ | 17 (18.1) | 7 (13.5) | 10 (23.8) |  |  |  |
| **Educational attainment** | | | |  | 0.1170^**^ |  |
| Senior high or secondary school | 4(4.3) | 3(5.8) | 1(2.4) |  |  |  |
| Junior college or College graduate | 62(65.9) | 38(73.1) | 24(57.1) |  |  |  |
| Graduate degree or higher | 28 (29.8) | 11 (21.1) | 17 (40.5) |  |  |  |
| **High-risk areas** | | | |  | 0.3336^**^ |  |
| No | 84 (89.4) | 48 (92.3) | 36 (85.7) |  |  |  |
| Yes | 10 (10.6) | 4 (7.7) | 6 (14.3) |  |  |  |
| **Pilot cities** | | | | 0.070 | 0.7919^*^ |  |
| No | 75 (79.8) | 42 (80.8) | 33 (78.6) |  |  |  |
| Yes | 19 (20.2) | 10 (19.2) | 9 (21.4) |  |  |  |
| **Economic level division** | | | | 0.799 | 0.6707^*^ |  |
| High GDP | 40 (42.6) | 20 (38.5) | 20 (47.6) |  |  |  |
| Medium GDP | 37 (39.4) | 22 (42.3) | 15 (35.7) |  |  |  |
| Low GDP | 17 (18.1) | 10 (19.2) | 7 (16.7) |  |  |  |
| **Commercial sexual behavior** | | | | 0.007 | 0.9345^*^ |  |
| No | 78 (83.0) | 43 (82.7) | 35 (83.3) |  |  |  |
| Yes | 16 (17.0) | 9 (17.3) | 7 (16.7) |  |  |  |
| **Monthly income(CNY)** | | | |  | 0.1349^**^ |  |
| No regular source of income | 60 (63.8) | 37 (71.1) | 23 (54.8) |  |  |  |
| <3000 RMB | 19 (20.2) | 8 (15.4) | 11 (26.2) |  |  |  |
| 3000-5000 | 10(10.6) | 3(5.7) | 7(16.7) |  |  | |
| 5000-8000 | 3(3.2) | 2(3.9) | 1(2.3) |  |  | |
| 8000~ | 2(2.2) | 2(3.9) | 0(0.0) |  |  | |
| **Results of the most recent HIV test during the preceding 6 months** | | | |  | 0.0367^**^ |  |
| Never | 4 (4.3) | 0 (0.0) | 4 (9.5) |  |  |  |
| Negative | 90 (95.7) | 52 (100.0) | 38 (90.5) |  |  |  |
| **STD** | | | |  | 1.0000^**^ |  |
| No | 84 (89.4) | 46 (88.5) | 38 (90.5) |  |  |  |
| Yes | 10(10.6) | 6(11.5) | 4(9.5) |  |  |  |
| **Sexual role with a man during the preceding 6 months** | | | |  | 0.0088^**^ |  |
| Top | 21 (22.3) | 11 (21.2) | 10 (23.8) |  |  |  |
| Versatile | 23 (24.5) | 19 (36.5) | 4 (9.5) |  |  |  |
| Bottom | 47 (50.0) | 20 (38.5) | 27 (64.3) |  |  |  |
| Oral | 3 (3.2) | 2 (3.8) | 1 (2.4) |  |  |  |
| **Frequency of condom use in anal sex during the preceding 6 months** | | | | 6.202 | 0.0128^*^ |  |
| Low | 30(31.9) | 11(21.2) | 19(45.2) |  |  |  |
| High | 64(68.1) | 41(78.9) | 23(54.8) |  |  |  |
| **Number of sexual partners during the preceding 6 months** | | | |  | 0.1565^**^ |  |
| 1-5 | 76 (80.9) | 40 (76.9) | 36 (85.7) |  |  |  |
| 6-10 | 11(11.7) | 9(17.3) | 2(4.8) |  |  |  |
| 11~ | 7(7.4) | 3(5.8) | 4(9.5) |  |  |  |
| **Multi-person sexual behavior** | | | | 1.473 | 0.2248^*^ |  |
| No | 68 (72.3) | 35 (67.3) | 33 (78.6) |  |  |  |
| Yes | 26 (27.7) | 17 (32.7) | 9 (21.4) |  |  |  |
| **Knowledge of HIV acquisition status of sexual partners in the last six months** | | | | 5.994 | 0.0499^*^ |  |
| Full | 45 (47.9) | 19 (36.5) | 26 (61.9) |  |  |  |
| Partial | 37 (39.4) | 25 (48.1) | 12 (28.6) |  |  |  |
| No | 12 (12.8) | 8 (15.4) | 4 (9.5) |  |  |  |
| **Substance use in the preceding 6 months** | | | | 4.506 | 0.0338^*^ |  |
| No | 38 (40.4) | 16 (30.8) | 22 (52.4) |  |  |  |
| Yes | 56(59.6) | 36(69.2) | 20(47.6) |  |  |  |
| **Ways of oral PrEP** | | | | 15.505 | 0.0004^*^ |  |
| Daily oral PrEP | 19 (20.2) | 6 (11.5) | 13 (31.0) | 68 |  |  |
| ED-PrEP | 48 (51.1) | 36 (69.2) | 12 (28.6) | 25 |  |  |
| Both | 27 (28.7) | 10 (19.2) | 17 (40.5) | 62 |  |  |

Note:*: χ^2^ test; **: Fisher's test

Table S4 Multi-factor logistic regression results for those who awareness of prep (stepwise regression method)

| **Variables** | **Ref** | ***β*** | **SE** | **Standardized *β*** | **Wald *χ^2^*** | ***P* value** | ***OR*（95%*CI*）** |
| --- | --- | --- | --- | --- | --- | --- | --- |
| **Results of the most recent HIV test during the preceding 6 months** | | | | | | | |
| Negative | vs. Never | 0.9888 | 0.2392 | 0.1691 | 17.0927 | <.0001 | 2.688(1.682,4.295) |
| **Knowledge of HIV acquisition status of sexual partners in the last six months** | | | | | | | |
| Partial | vs. Full | -0.1718 | 0.2215 | -0.0448 | 0.6016 | 0.4380 | 0.842(0.546,1.300) |
| No |  | -0.6024 | 0.2401 | -0.1257 | 6.2960 | 0.0121 | 0.547(0.342,0.876) |
| **Frequency of condom use in anal sex during the preceding 6 months** | | | | | | | |
| High | vs. Low | 0.4708 | 0.1995 | 0.1157 | 5.5681 | 0.0183 | 1.601(1.083,2.367) |

Table S5 Multi-factor logistic regression results for those who are willing to take prep (stepwise regression method)

| **Variables** | **Ref** | ***β*** | **SE** | **Standardized *β*** | **Wald *χ^2^*** | ***P* value** | ***OR*（95%*CI*）** |
| --- | --- | --- | --- | --- | --- | --- | --- |
| **Pilot cities** | | | | | | | |
| Yes | vs. No | 0.7389 | 0.2996 | 0.1089 | 6.0834 | 0.0136 | 2.094(1.164,3.766) |
| **Results of the most recent HIV test during the preceding 6 months** | | | | | | | |
| Negative | vs. Never | 0.4958 | 0.2272 | 0.0787 | 4.7636 | 0.0291 | 1.642(1.052,2.563) |
| **Sexual role with a man during the preceding 6 months** | | | | | | | |
| Top | vs. Bottom | -0.5120 | 0.1620 | -0.1264 | 9.9838 | 0.0016 | 0.599(0.436,0.823) |
| Versatile |  | -0.1879 | 0.1900 | -0.0411 | 0.9783 | 0.3226 | 0.829(0.571,1.203) |
| Oral |  | 0.00837 | 0.2511 | 0.00133 | 0.0011 | 0.9734 | 1.008(0.616,1.650) |
| **Substance use in the preceding 6 months** | | | | | | | |
| Yes | vs. No | 0.5589 | 0.1484 | 0.1501 | 14.1740 | 0.0002 | 1.749(1.307,2.339) |

Note: Adjusted for the following covariates: Economic level division, Commercial sexual behavior, STDs, Multi-person sexual behavior, and HIV status of sexual partners.

Table S6 Multi-factor logistic regression results for those actually taking prep (stepwise regression method)

| **Variables** | **Ref** | ***β*** | **SE** | **Standardized *β*** | **Wald *χ^2^*** | ***P* value** | ***OR*（95%*CI*）** |
| --- | --- | --- | --- | --- | --- | --- | --- |
| **Educational attainment** | | | | | | | |
| Junior college or College graduate | vs. Senior high or secondary school | 0.6335 | 0.5585 | 0.1522 | 1.2865 | 0.2567 | 1.884(0.631,5.630) |
| Graduate degree or higher |  | 1.4444 | 0.5897 | 0.3056 | 5.9982 | 0.0143 | 4.239(1.334,13.467) |
| **High-risk areas** | | | | | | | |
| Yes | vs. No | 1.6258 | 0.4216 | 0.1926 | 14.873 | 0.0001 | 5.082(2.224,11.612) |
| **Pilot cities** | | | | | | | |
| Yes | vs. No | 1.0263 | 0.3173 | 0.1652 | 10.46 | 0.0012 | 2.791(1.498,5.198) |
| **Multi-person sexual behavior** | | | | | | | |
| Yes | vs. No | 0.7821 | 0.2911 | 0.1488 | 7.2198 | 0.0072 | 2.186(1.236,3.867) |
| **Substance use in the preceding 6 months** | | | | | | | |
| Yes | vs. No | 0.6461 | 0.2506 | 0.1767 | 6.6452 | 0.0099 | 1.908(1.167,3.118) |

Note: Adjusted for the following covariates: age, Commercial sexual behavior, etc.

Table S7 Multi-factor logistic regression results for those who adhered to prep (stepwise regression method)

| **Variables** | **Ref** | ***β*** | **SE** | **Standardized *β*** | **Wald *χ^2^*** | ***P* value** | ***OR*（95%*CI*）** |
| --- | --- | --- | --- | --- | --- | --- | --- |
| **Sexual role with a man during the preceding 6 months** | | | | | | | |
| Top | vs. Bottom | -0.9222 | 0.7035 | -0.2129 | 1.7182 | 0.1899 | 0.398(0.100,1.579) |
| Versatile |  | -2.1636 | 0.7328 | -0.5156 | 8.7175 | 0.0032 | 0.115(0.027,0.483) |
| Oral |  | -1.8041 | 1.4686 | -0.1758 | 1.509 | 0.2193 | 0.165(0.009,2.928) |
| **Frequency of condom use in anal sex during the preceding 6 months** | | | | | | | |
| High | vs. Low | -1.6841 | 0.64 | -0.4351 | 6.9232 | 0.0085 | 0.186(0.053,0.651) |
| **Substance use in the preceding 6 months** | | | | | | | |
| Yes | vs. No | -1.4969 | 0.6028 | -0.4072 | 6.1658 | 0.0130 | 0.224(0.069,0.730) |
| **Ways of oral PrEP** | | | | | | | |
| Daily oral PrEP | vs. ED-PrEP | 1.9551 | 0.727 | 0.4352 | 7.2324 | 0.0072 | 7.065(1.699,29.371) |
| Both |  | 2.0274 | 0.6609 | 0.5085 | 9.4106 | 0.0022 | 7.594(2.079,27.736) |

Note: Adjusted for the following covariates: most recent HIV test result, HIV acquisition status of sexual partners in the last 6 months, etc.
